# Adherence to a Mediterranean Diet Is Associated with Lower Depressive Symptoms among U.S. Adults

**DOI:** 10.3390/nu14020278

**Published:** 2022-01-11

**Authors:** Vanessa M. Oddo, Lauren Welke, Andrew McLeod, Lacey Pezley, Yinglin Xia, Pauline Maki, Mary Dawn Koenig, Michelle A. Kominiarek, Scott Langenecker, Lisa Tussing-Humphreys

**Affiliations:** 1 Department of Kinesiology and Nutrition, University of Illinois at Chicago, 1919 W Taylor, Chicago, IL 60612, USA; voddo@uic.edu (V.M.O.); lwissl2@uic.edu (L.P.); 2 AbbVie, 1 N Waukegan Rd., Chicago, IL 60064, USA; laurenwelke@gmail.com; 3 Institute for Health Research and Policy, University of Illinois at Chicago, 1747 W Roosevelt Rd., Chicago, IL 60612, USA; amcleo2@uic.edu; 4 Department of Medicine, University of Illinois at Chicago, 1853 W Polk St., Chicago, IL 60612, USA; yxia@uic.edu; 5 Department of Psychology, University of Illinois at Chicago, 1007 W Harrison St., 1009 BSB, Chicago, IL 60612, USA; pmaki1@uic.edu; 6 Department of Psychiatry, University of Illinois at Chicago, 1601 W Taylor St., Chicago, IL 60612, USA; 7 Department of Human Development Nursing Science, University of Illinois at Chicago, 845 S Damen Ave., Chicago, IL 60612, USA; marydh@uic.edu; 8 Department of Obstetrics and Gynecology, Northwestern University Clinical and Translational Sciences Institute, 250 E Superior St., Chicago, IL 60611, USA; michelle.kominiarek@nm.org; 9 Department of Psychiatry, University of Utah, 383 Colorow Way, Salt Lake City, UT 84018, USA; s.langenecker@hsc.utah.edu; 10 Cancer Center, University of Illinois at Chicago, 818 S Wolcott Ave., Chicago, IL 60612, USA

**Keywords:** alternate Mediterranean diet score, depressive symptoms, Mediterranean diet, mental health, dietary patterns

## Abstract

Depression is a leading cause of disability, yet current prevention and treatment approaches have only had modest effects. It is important to better understand the role of dietary patterns on depressive symptoms, which may help prevent depression or complement current treatments. This study examined whether adherence to a Mediterranean diet (Med Diet), determined by the Alternate Med Diet score (aMED), was associated with depressive symptoms in a representative sample of U.S. adults. The aMED score (range 0–9) was calculated from a 24-h diet recall with gender-specific quartiles (Q) estimated. The Patient Health Questionnaire-9 (PHQ-9) was used to define depressive symptoms, which was dichotomized as no to mild (0–9) versus moderate to severe symptoms (10–27). Logistic regression was used to investigate the association between quartiles of aMED and depressive symptoms when controlling for sociodemographics, total calories, and the time of year of diet recall; 7.9% of the sample had moderate to severe depressive symptoms. Compared to individuals with the lowest aMED (Q1), individuals in Q3 and Q4 had 40% and 45% lower odds of moderate to severe depressive symptoms (odds ratio [OR] = 0.60, 95% confidence interval [CI]: 0.50, 0.74; OR = 0.55, 95% CI: 0.36, 0.84, respectively). This study provides modest support of Med Diet’s role in supporting positive mental health.

## 1. Introduction

In 2019, 19.4 million adults or 7.8% of the United States (U.S.) adult population experienced a major depressive episode [1]. Depression is a leading cause of disability [2] and is estimated to cost the U.S. $210 billion annually [3]. Current prevention and treatment approaches for depression have had modest effects [4,5] and pharmaceutical approaches for treating depression have associated side effects [6,7]. At the same time, some prior evidence finds that adhering to a largely plant-based dietary pattern may play an important role in mental health [8]. Thus, it is important to better understand diet’s role in depression onset and treatment.

The etiology of depression is likely a contribution of social, genetic, environmental, psychological, and biochemical factors [9]. Lifestyle factors including diet can contribute to hypothalamic-pituitary-adrenal axis dysfunction, oxidative stress, and inflammatory pathway activation that may promote the onset and chronicity of depression [10]. Micronutrient deficiency studies demonstrate the importance of nutrition in maintaining mental health [11]. Essential fatty acids, including omega-3 polyunsaturated fatty acids (PUFAs), play an important role in quelling brain inflammation and optimizing brain vascular health, both of which may be integral to maintaining mood [12]. Antioxidant nutrients including vitamins C and E, selenium and carotenoids (e.g., lutein, zeaxanthin, β-carotene) may also play an important role in maintaining mental health and mood by counteracting oxidative stress [11,13]. Folate has been linked to brain function, mental health, and mood, and is required for the metabolism and synthesis of neurotransmitters including serotonin that are essential to healthy central nervous system function [14]. However, humans do not consume single nutrients, rather meals that contain a multitude of nutrient exposures. Accordingly, it is important to understand the role of dietary patterns on mental health.

Some recent evidence supports that pro-inflammatory dietary patterns are associated with greater risk of depressive symptoms [15]. Conversely, adhering to a largely plant-based dietary pattern may play an important role in preventing depression and reducing depressive symptoms [16,17]. Specifically, a Mediterranean Diet (Med Diet), characterized by a high intake of fruits, vegetables, legumes, whole grains, fish, nuts, low-fat dairy and olive oil, has been associated with decreased risk of depression in non-U.S. populations [17,18]. The positive mental health benefits associated with a Med Diet are believed to be due, in part, to the synergistic effect of high monounsaturated fatty acids, fiber and antioxidants on the gut microbiome and host oxidative stress and pro-inflammatory pathways [19,20].

We build upon prior literature showing a relationship between Med Diet and mood [13,21] by investigating the relationship between adherence to a Med Diet and depressive symptoms in a nationally representative sample of U.S. adults 20 years and older. It is important to understand the role of dietary patterns on depressive symptoms, as adherence to a more healthful dietary pattern may be an effective strategy to prevent and/or mitigate depressive symptoms in U.S. adults and serve as a complement to the current psychiatric and pharmacological treatments.

## 2. Materials and Methods

Data were obtained from the nationally representative National Health and Nutrition Examination Survey (NHANES) from 2007–2012 [22]. NHANES is conducted in 2-year cycles, with data collection occurring during in-home interviews and in mobile examination centers. These analyses utilized sociodemographic, anthropometric, lifestyle behavior, biochemical, health status, and dietary intake (i.e., 24-h diet recall) data from three survey waves.

### 2.1. Subjects

Our original sample included 30,442 individuals from the 2007–2012 survey waves. Individuals were first excluded from this analysis if they were less than 20 or greater than 79 years old (*n* = 13,956). We then excluded individuals that were pregnant (*n* = 180), missing data on day 1 dietary intake (*n* = 1,446), missing depression survey items (*n* = 1070), or had an implausible energy intake data (*n* = 993) (<500 kcals or >3500 kcals for women and <800 kcals or >4200 kcals for men) [23]. Individuals were also excluded if they were missing data for our primary covariates of interest (described below) (*n* = 1028). Our analytic sample included 11,769 adults.

### 2.2. Independent Variable: Mediterranean Diet Adherence Score

Dietary intake was assessed via interviewer administered 24-h diet recall. One diet recall was obtained during the in-person assessment in the mobile examination center (day 1) and the second over the phone (day 2) within 10 days of the in-person assessment. For our primary analyses, we used the 24-h diet recall obtained during the in-person interview (day 1 recall), because in each 2-year cycle, approximately 20–30% of subjects fail to complete the day two diet recall [24].

Adherence to a Med Diet was determined in two steps. First, the 24-h diet recall data was linked to the United States Department of Agriculture (USDA) Food Patterns Equivalents Databases to convert the foods and beverages to USDA food patterns equivalents [25]. Second, a Med Diet score was calculated using the alternate Mediterranean Diet score (aMED) [26], modified for use with 24-h diet recall data by Gaskins and colleagues [27]. aMED includes nine components: vegetables, legumes, fruits, nuts, whole grains, red and processed meats, fish, alcohol, and the ratio of monounsaturated to saturated fats. For purposes of these analyses, gender-specific median cut-points and aMED total and component scores were calculated [28]. One point was awarded if the individual consumed above the sex-specific group median for vegetable, fruit, legumes, whole grain, nut, and fish intake and a monounsaturated/saturated fat ratio, and one point was awarded if an individual consumed at or below the sex-specific median intake for red and processed meats. For alcohol, 1 point was awarded for consumption of 5–25 g of ethanol per day for women, and 10–50 g of ethanol for men, indicating moderate intake, and 0 points were awarded for consumption outside of these ranges. The aMED score ranged from 0–9 points, with higher scores indicating greater adherence to a Med Diet. We then estimated quartiles (Q) of the aMED score, which served as our primary independent variable.

### 2.3. Dependent Variable: Depression

Depressive symptoms were measured using the Patient Health Questionnaire-9 (PHQ-9) [29], which is a nine-item screening instrument that probes for frequency of depressive symptoms experienced during the previous 2 weeks and is a reliable and valid measure of depression in adults [29,30]. For each of the nine questions, subjects responded using a four-point Likert scale, with response options ranging from not at all (0), several days (1), more than half the days (2), and nearly every day (3) (score range 0–27 points). Using the PHQ-9 scores, depression severity was dichotomized as no or mild (0–9) versus moderate or severe (10–27) using established cut-points [29].

### 2.4. Covariates and Effect Modifiers

We used a directed acyclic graph to identify factors that might affect both diet quality and depression symptoms, including sociodemographics (age, gender, race/ethnicity, educational attainment, relationship status, and family income to poverty ratio), total caloric intake and time of year of dietary recall (November–April or May–October). Additionally, we hypothesized the effect of aMED on depression might vary by gender, age, and/or body mass index (BMI).

### 2.5. Statistical Analysis

Descriptive statistics for continuous variables were estimated as the mean ± standard error. We calculated frequency and proportion for categorical variables. We also described nutrient intakes based on the one 24-h diet recall; we estimated the contribution of carbohydrates, fats, and proteins as the percent of total calories and other nutrients are presented per 1000 kcal consumed. We used multivariable logistic regression to estimate the odds of depression across the aMED quartiles when controlling the aforementioned covariates.

Additionally, we tested whether high (versus low) aMED scores, varied by gender, age, and BMI with the inclusion of an interaction term (e.g., high aMED X gender). Higher Med Diet adherence was defined as greater than the median total aMED score (i.e., 3). The interaction terms for age and BMI were statistically significant. Therefore, we also present results stratified by age categories (<30 years old, 30–55 years old, >55 years old) and BMI (normal weight, overweight defined as BMI ≥ 25 kg/m^2^ and BMI <30 kg/m^2^, and obesity defined as BMI ≥ 30 kg/m^2^).

### 2.6. Sensitivity Analyses

In sensitivity analyses, we additionally controlled for (1) health conditions (BMI and self-reported hypertension, hypercholesterolemia, diabetes, cardiovascular diseases, liver diseases, cancers); (2) lifestyle behaviors (currently smoking, recent alcohol consumption, minutes of moderate to vigorous physical activity over the past 30 days); (3) day of diet collection (e.g., Monday); and (4) systemic inflammation measured via C-reactive protein (CRP). Additionally, we utilize day two of the 24-h diet recall data. Utilizing two days of diet data, we estimate the effect of a Med Diet on depressive symptoms when (1) using the mean value for aMED components between day 1 and day 2; (2) estimating intake using the population ratio method (https://epi.grants.cancer.gov/hei/population-ratio-method.html (accessed on 3 November 2021)); and (3) estimating intake when using the bivariate method (https://epi.grants.cancer.gov/hei/bivariate-method.html (accessed on 3 November 2021)). The population ratio method estimates the ratios of the dietary constituents to 1000 kcal of energy, with the exception of fatty acids, which use the ratio of the sum of monounsaturated fatty acids to saturated fatty acids and then each ratio is scored based on the median value as described above. The bivariate method uses adaptive Gaussian quadrature to predict usual intake for each individual and then employs the aforementioned scoring standards for each component (i.e., using the gender-specific group medians).

All statistical analyses were performed using Stata version 15.1 (StataCorp LLC, College Station, TX, USA) and used survey design procedures to account for NHANES sampling, stratification and clustering methods. Six-year sampling weights (2007–2008, 2009–2010, and 2011–2012) were calculated by multiplying the sample weight provided by NHANES for each 2-year cycle by one-third. Alpha was set to 0.05. The current analysis was deemed exempt from human subjects research regulatory requirements by the University of Illinois Chicago’s Institutional Review Board.

## 3. Results

### 3.1. Descriptive

Survey weighted descriptive statistics are presented in Table 1. Aligned with the U.S. population in the years surveyed by design, about half of the sample was female (51.6%) and individuals were aged 46.2 years (standard error [SE] = 0.4), on average. Approximately 15% of the sample were lower-income, 56.3% were married and one-third had a college-level of education (30.0%). A majority of the sample (70.4%) was non-Hispanic White; 12.8% of the weighted sample were Hispanic, 10.7% were non-Hispanic Black, and 6.1% were individuals of another race/ethnicity.

The average PHQ-9 score was 3.0 points (SE = 0.1) and 7.9% of the sample had moderate to severe depressive symptoms. One-third of adults surveyed had hypertension, nearly 40% had hypercholesterolemia and the average BMI was 28.9 kg/m^2^ (SE = 0.1). Smaller proportions of the sample had cardiovascular conditions (7.2%), type 2 diabetes (8.8%), liver conditions (3.1%) and cancer (8.8%).

Individuals with the highest aMED score (Q4) compared to the lowest (Q1) were somewhat older, less likely to be below the poverty threshold (6.9% of Q4 versus 20.0% of Q1) and had higher levels of educational attainment (54.7% of Q4 were college educated, compared to 18.2% of Q1). Persons in aMED Q4 also reported fewer depressive symptoms on the PHQ-9 (2.3 vs. 3.7 points) compared to those in Q1; PHQ-9 decreased with increasing aMED score. Those in aMED Q4 were also less likely to report moderate to severe depressive symptoms (4.0% vs. 11.2%), had lower BMIs (27.1 vs. 29.7 kg/m^2^) and had lower CRP (0.2 vs. 0.3 mg/dL).

Food-based nutrient intakes are presented in Table 2. Persons in the highest aMED quartile (Q4) compared to the lowest (Q1), had higher intake of total calories (2198.2 versus 2029.6 kcal), but a lower percentage of calories from total fat (32.9% versus 34.0%) and saturated fat (8.8% versus 12.3%). Persons with the highest adherence had higher consumption of dietary fiber (11.9 versus. 5.6 gm per 1000 kcal), PUFAs (9.9 versus 8.4 gm per 1000 kcal) and most micronutrients, including folate, iron, and calcium.

### 3.2. Regression-Based Estimates

The adjusted association between aMED and depressive symptoms is detailed in Table 3. Compared to individuals in Q1, individuals with higher aMED scores had a 40% (Q3 odds ratio [OR] = 0.60; 95% confidence interval [CI]: 0.50, 0.74) to 45% (Q4 OR = 0.55; 95% CI: 0.36, 0.84) lower odds of moderate to severe depressive symptoms.

We observed some heterogeneity in the association by age and BMI (Table 4). We did not observe a significant association between aMED and depressive symptoms among individuals aged <30 years (OR = 1.02; 95% CI: 0.60, 1.75). However, among individuals aged 30–55 years, higher versus lower aMED score was associated with 38% lower odds of moderate to severe depressive symptoms (OR = 0.62; 95% CI: 0.49, 0.80). Among individuals aged >55 years, higher aMED score was associated with 54% lower odds of moderate to severe depressive symptoms (OR = 0.46; 95% CI: 0.33, 0.64). Among normal weight individuals, higher (versus lower) aMED score was associated with 43% lower odds of moderate to severe depressive symptoms (OR = 0.57; 95% CI: 0.38, 0.86). Higher aMED score was associated with 34% (OR = 0.66; 95% CI: 0.43, 1.00) and 29% (OR= 0.71; 95% CI: 0.55, 0.90) lower odds of moderate to severe depressive symptoms among individuals with overweight and obesity, respectively.

### 3.3. Sensitivity Analyses

Our primary results among Q3 and Q4 were similar in magnitude and statistical significance when also controlling for health conditions (OR = 0.48; 95% CI: 0.31, 0.73), day of data collection (OR = 0.55; 95% CI: 0.37, 0.84) and systematic inflammation (OR = 0.47; 95% CI: 0.25, 0.90) (Table 3). However, results were attenuated and no longer significant among Q4 when controlling for lifestyle behaviors (OR: 0.70; 95% CI: 0.45, 1.07).

Our primary results were also similar in magnitude and statistical significance when we included the second day of 24-h dietary intake and (1) used the average value between day 1 and day 2 (OR = 0.49; 95% CI: 0.35, 0.69) and (2) when estimating intake using the population ratio method (OR = 0.45; 95% CI: 0.32, 0.63) (Table 5). Results were not statistically significant when using the bivariate method to predict usual intake (OR = 1.03; 95% CI: 0.84, 1.27).

## 4. Discussion

We evaluated the association between adherence to a Med Diet and depressive symptoms among a large representative sample of U.S. adults. In this sample, 8% of individuals endorsed moderate to severe depressive symptoms based on the PHQ-9. We found that greater adherence to a Med Diet was associated with 40–45% lower odds of moderate to severe depressive symptoms, which provides modest support for the diet’s role in mental health [18,31,32,33]. However, we observed an attenuation of the results among the most adherent (Q4) when adjusting for lifestyle behaviors (i.e., smoking status, recent alcohol use, and physical activity) as well as when using the bivariate method to predict usual dietary intake, which is a more conservative approach to estimating dietary intake. This suggests that the protective effect of a Med Diet on depressive symptoms may be driven by other healthful lifestyle behaviors associated with a healthful diet, such as decreased smoking rates and increased physical activity, as seen in other studies [34].

It is difficult to directly compare our study to others, as no other investigators have used aMED to examine associations with depressive symptoms. aMED uses a scoring approach driven by the median intake values of the analytical sample whereas a Med Diet scoring index like that developed by Panagiotakos et al. is based on pre-defined consumption patterns allowing for more direct comparison across studies [35]. Nevertheless, our results are generally consistent with prior studies. In the SUN cohort study, which used the original Med Diet score [36], the median score in the most adherent quantile was 6 points, the same score as our most adherent group (Q4). Among this cohort of 15,093 Spanish middle-aged educated adults free of depression at baseline, those with moderate to high adherence to a Med Diet had 25–30% lower risk of developing depression at 10-year follow-up [17]. In a cross-sectional analysis of U.S. men and women (NHANES 2007–2010), high adherence to a “healthy” dietary pattern, similar to a Med Diet consisting of whole grains, vegetables, fruits, fish, nuts, and seeds, was associated with 23% reduced odds of depression in men, and 40% reduced odds in women, compared to their less “healthy” counterparts [37]. Similarly, a cross-sectional analysis of 8369 women participating in the Australian Longitudinal Study on Women’s Heath found that those adhering to a Med Diet, had 18% lower odds of depression, compared to those who did not follow a Med Diet [38]. In the Prevención con Dieta Mediterránea (PREDIMED) study, researchers found no effect of a Med Diet supplemented with tree nuts or olive oil on depression risk (compared to a low-fat control diet) at three-year follow-up [18]. However, the authors did find that in those with type 2 diabetes assigned to a Med Diet, there was a 41% lower risk of depression compared to low-fat controls. This suggests that those who might benefit most from a Med Diet are those with underlying metabolic dysfunction (i.e., insulin resistance) and a heightened inflammatory state, which are both hallmarks of type 2 diabetes, and known risk factors for depression [39,40,41].

In our study, we saw a stronger relationship between aMED and depressive symptoms with increasing age but not among individuals with obesity. Advanced age and obesity are positively related to insulin resistance and a heightened inflammatory state [42,43]. In line with the findings from PREDIMED, our results for age show that with increasing age, the odds of having depressive symptoms when adhering to a Med Diet decreases. However, we did not see the same effect with increasing BMI category. This was a somewhat surprising result; however, among the individuals with obesity, overall Med Diet adherence was lower when compared to individuals with normal weight and overweight and this may have attenuated the strength of the relationship between Med Diet adherence and depressive symptoms. The finding that Med Diet adherence is lower among adults with obesity is in concordance with a recent paper examining Med Diet adherence among adults residing in the Middle East [44].

Within the existing observational studies and RCTs, biological mechanisms that mediate the association between Med Diet adherence and reduced depressive symptoms and depression risk have not been fully examined. However, there are several plausible mechanisms including reduced inflammation and oxidative stress. Like the other non-interventional studies, we observed lower circulating CRP, a pro-inflammatory biomarker, among those with greater Med Diet adherence [26,45,46,47,48]. The inflammation lowering effect of a Med Diet may positively influence mood by reducing oxidative damage products [49], increasing total antioxidant capacity and antioxidant levels [49], normalizing neurotransmitter production, HPA axis function and glucocorticoid receptor signaling [14,50,51,52,53,54] all of which are altered with depression [55,56,57]. Moreover, in our analysis, those closely adhering to a Med Diet had higher intakes of dietary fiber, PUFAs and vitamins and minerals from whole foods and beverages. Fiber, a key component of fruits, vegetables, and whole grains, may suppress inflammation through its effect on glucose metabolism [20] and its stimulation of the gut microbiota to promote their production of anti-inflammatory metabolites, including the short chain fatty acid butyrate [58]. Omega-3 PUFAs may lower inflammation and benefit mood by fostering the production of anti-inflammatory prostaglandin E3 versus pro-inflammatory prostaglandin E2, lowering postprandial triglycerides, increasing chylomicron triglyceride clearance, and reducing oxidation of lipids [59,60,61]. Vitamins C and E, prominent in a Med Diet, also have antioxidant properties that lower oxidative stress and inflammation [62]. Therefore, the Med Diet’s anti-inflammatory and mood-related benefits may be attributed to the synergy of nutrients that comprise the dietary pattern.

This study has several limitations. First, due to the cross-sectional design, we were unable to espouse a causal relationship between Med Diet adherence and depressive symptoms, and there is the possibility for residual confounding by factors unobserved in our data. However, we used a large, nationally representative sample, making our results more generalizable compared to smaller studies conducted in singular geographic locations. Second, diet data were calculated from 24-h diet recalls, and it is known that self-report dietary data are associated with underreporting and misreporting given that they rely on memory and estimation of food quantities by the participant [63]. Third, although NHANES is designed so that a single 24-h recall can be used to estimate the mean consumption of a population, using only a single 24-h recall in our primary analysis may not best capture usual intake. Fourth, prescription drugs, current use of psychotherapy and dietary supplement use was not included in these analyses and are exposures that could have differential effects on reporting of depressive symptoms and eating behavior. Finally, although the aMED score used to measure Med Diet adherence is widely used in the nutritional epidemiologic literature, it is a score based on the median intake values of the population being studied; this makes comparison to other populations difficult and may not be the most accurate reflection of adherence to a Med Diet.

## 5. Conclusions

Greater adherence to a Med Diet was associated with lower moderate to severe depressive symptoms in a representative sample of U.S. adults. Thus, we provide modest evidence suggesting that a Med Diet may support the prevention and treatment of depression in adults. Future directions should include controlled dietary intervention studies in diverse adult populations designed to examine the effect of a Med Diet on depressive symptoms and depression. These studies should also incorporate the assessment of potential biological mediators including inflammation, HPA axis function, oxidative stress, and the gut microbiome to elucidate mechanisms through which a Med Diet affects mood.

## Figures and Tables

**Table 1:** Selected characteristics of adult men and women overall and by Alternate Mediterranean Diet (aMED) score quartiles, National Health and Nutrition Examination Survey (NHANES) 2007–2012.

	N (%) or Mean (Standard Error) ^1^				
	Overall (*n* = 11,769)	Q1 aMED, 0–2 pts (*n* = 3621)	Q2 aMED, 3 pts (*n* = 2689)	Q3 aMED, 4–5 pts (*n* = 4155)	Q4 aMED, 6–9 pts (*n* = 1304)
Sociodemographic					
Females	5989 (51.6%)	1919 (53.3%)	1342 (51.0%)	2126 (51.5%)	602 (48.6%)
Mean age (years)	46.2 (0.4)	43.1 (0.5)	45.8 (0.4)	47.6 (0.5)	50.0 (0.6)
Below PIR ^2^	2579 (14.6%)	1024 (20.0%)	651 (16.1%)	758(11.7%)	14 (6.9%)
Relationship status					
Married	6123 (56.3%)	1679 (50.6%)	1308 (52.4%)	2319 (59.8%)	817 (66.9%)
Divorced/Separated/Widowed	2503 (17.3%)	815 (18.9%)	613 (19.3%)	868 (16.6%)	207 (11.9%)
Never married	2227 (18.8%)	794 (21.7%)	541 (20.4%)	687 (16.6%)	205 (14.9%)
Living with partner	916 (7.6%)	333 (8.8%)	227 (7.9%)	281 (6.9%)	75 (6.3%)
Educational attainment					
<Highschool	2963 (16.5%)	1109 (21.7%)	749 (18.9%)	934 (13.7%)	171 (7.6%)
High school graduate or GED	2689 (22.4%)	954 (27.5%)	681 (25.1%)	870 (20.0%)	184 (12.4%)
Some college or associates degree	3420 (31.1%)	1062 (32.7%)	771 (31.4%)	1244 (31.6%)	343 (25.3%)
≥College graduate	2697 (30.0%)	496 (18.2%)	488 (24.7%)	1107 (34.8%)	606 (54.7%)
Race/ethnicity					
Non-Hispanic White	5368 (70.4%)	175 (70.7%)	1152 (67.7%)	1802 (69.6%)	656 (77.1%)
Hispanic	2976 (12.8%)	851 (12.7%)	741 (14.6%)	1118 (13.2%)	266 (8.4%)
Non-Hispanic Black	2529 (10.7%)	848 (12.7%)	612 (11.6%)	872 (10.1%)	197 (6.0%)
Other	896 (6.1%)	164 (3.9%)	184 (6.1%)	363 (7.1%)	185 (8.5%)
Lifestyle Behaviors					
Currently smoking	3114 (26.1%)	1349 (37.4%)	769 (28.3%)	824 (19. 4%)	172 (13.9%)
Recent alcohol use	2921 (28.2%)	670 (20.3%)	591 (24.0%)	1136 (31.1%)	524 (47.3%)
Mean minutes moderate physical activity (weekly)	96.9 (2.6)	86.2 (4.0)	89.9 (4.6)	101.7 (3.8)	121.8 (6.5)
Health					
Mean PHQ-9 score (range 0–27)	3.0 (0.1)	3.7 (0.1)	3.3 (0.1)	2.6 (0.07)	2.3 (0.1)
Moderate/severe depression (10–27)	1125 (7.9%)	463 (11.2%)	299 (9.5%)	303 (5.5%)	60 (4.0%)
Mean BMI (kg/m^2^)	28.9 (0.11)	29.8 (0.14)	29.3 (0.16)	28.5 (0.2)	27.1 (0.21)
Mean CRP (mg/dL) ^3^	0.3 (0.0)	0.3 (0.0)	0.3 (0.0)	0.3 (0.0)	0.2 (0.0)
Hypertension	4092 (30.2%)	1219 (29.3%)	950 (31.0%)	1495 (31.6%)	428 (27.3%)
Hypercholesterolemia	3823 (38.8%)	1051(36.6%)	866 (40.7%)	1416 (39.5%)	490 (38.1%)
Cardiovascular conditions ^4^	1101 (7.2%)	355 (6.8%)	243 (7.4%)	410 (8.0%)	93 (5.9%)
Type 2 diabetes	1421 (8.8%)	418 (8.3%)	338 (10.2%)	543 (9.2%)	122 (6.6%)
Liver condition	424 (3.1%)	126 (2.6%)	89 (2.8%)	150 (3.2%)	59 (4.3%)
Cancer	1016 (8.8%)	269 (7.2%)	206 (8.9%)	389 (9.6%)	152 (10.4%)

aMED = Alternate Mediterranean Diet score; CRP = C-reactive protein; BMI = body mass index; PHQ-9 = Patient Health Questionnaire 9; PIR = poverty-to-income ratio; Q = Quartile. ^1^ Ns are unweighted. Percentages, means and standard errors are weighted to account for NHANES sampling, stratification and clustering. ^2^ Poverty-to-income ratio < 1. ^3^ CRP values are log transformed. ^4^ Includes stroke, heart attack, angina, congestive heart failure, and coronary heart disease.

**Table 2:** Nutrient intake from one 24-h diet recall for adult men and women overall and by Alternate Mediterranean Diet score (aMED) quartile, NHANES 2007–2012.

	% or Mean (Standard Error) ^1^				
	Overall (*n* = 11,769)	Q1 aMED (*n* = 3621)	Q2 aMED (*n* = 2689)	Q3 aMED (*n* = 4155)	Q4 aMED (*n* = 1304)
Macronutrients					
Energy (kcal)	2087.9 (10.7)	2029.6 (17.1)	2052.7 (20.1)	2121.5 (15.0)	2198.2 (29.7)
% kcals from carbohydrate	48.9% (0.2)	47.9 % (0.3)	48.6% (0.3)	49.9% (0.3)	49.4% (0.2)
% kcal from protein	15.7% (0.1)	15.5% (0.1)	15.7% (0.1)	15.7% (0.1)	15.8% (0.2)
% kcals from total fat	33.4% (0.1)	34.0% (0.3)	33.6% (0.3)	33.0% (0.2)	32.9% (0.3)
% kcals from saturated fat	10.8% (0.1)	12.3% (0.1)	11.0% (0.1)	10.2% (0.1)	8.8% (0.1)
MUFA per 1000 kcal (gm)	13.4 (0.1)	13.4 (0.1)	13.4 (0.1)	13.3 (0.1)	13.9 (0.2)
PUFA per 1000 kcal (gm)	8.4 (0.1)	7.3 (0.1)	8.3 (0.1)	8.9 (0.1)	9.9 (0.1)
Micronutrients per 1000 kcal					
Fiber (gm)	8.2 (0.1)	5.6 (0.1)	7.7 (0.1)	9.6 (0.1)	11.9 (0.2)
Cholesterol (mg)	134.0 (1.3)	145.2 (2.3)	139.2 (1.8)	129.35 (1.7)	111.3 (3.2)
Sodium (mg)	1684.9 (5.8)	1688.0 (11.7)	1695.0(13.2)	1695.6 (9.5)	1628.9 (15.9)
Folate (mcg)	200.2 (1.6)	167.7 (1.8)	193.8 (2.3)	218.0(2.1)	240.6 (4.2)
Iron (mg)	7.4 (0.1)	6.7 (0.1)	7.3(0.1)	7.8 (0.1)	8.0 (0.1)
Calcium (mg)	468.2 (3.8)	465.0 (6.1)	454.6 (6.5)	477.0 (5.0)	475.6 (8.4)
Vitamin C (mg)	41.7 (1.0)	27.2 (1.3)	40.5 (1.4)	48.8 (1.3)	58.9 (1.7)
Vitamin K (mcg)	56.8 (2.2)	33.1 (1.4)	50.1 (2.05)	71.2 (5.2)	86.0(4.4)
Vitamin E (mg)	3.9 (0.1)	2.9 (0.1)	3.6 (0.1)	4.3 (0.1)	5.5 (0.1)
Vitamin B12 (mcg)	2.5 (0.1)	2.5 (0.1)	2.4 (0.1)	2.7 (0.1)	2.5 (0.1)
Thiamin (Vit B1) (mg)	0.8 (0.0)	0.7 (0.0)	0.8 (0.0)	0.8 (0.0)	0.9 (0.0)
Riboflavin (Vit B2) (mg)	1.1 (0.0)	1.0 (0.0)	1.0 (0.0)	1.1 (0.05)	1.1 (0.0)
Magnesium (mg)	147.4 (1.1)	119.9(0.7)	139.9 (1.3)	160.1(1.37)	192.0 (2.1)
Phosphorus (mg)	657.5 (2.9)	635.6 (3.9)	646.2 (4.8)	670.9 (4.8)	693.7(6.3)
Selenium (mcg)	53.4 (0.3)	52.5 (0.4)	52.3 (0.4)	53.9 (0.5)	55.9 (1.0)
Zinc (mg)	5.6 (0.0)	5.6 (0.1)	5.6 (0.1)	5.7 (0.1)	5.5 (0.1)

Gm = grams; Kcal = kilocalorie; Mcg = micrograms; Mg = milligrams; MUFA = monounsaturated fatty acids; PUFA = polyunsaturated fatty acids. ^1^ Values are weighted to account for NHANES sampling, stratification and clustering.

**Table 3:** Association between Alternate Mediterranean Diet score (aMED) and moderate to severe depressive symptoms, NHANES 2007–2012.

	Odds Ratio (95% Confidence Interval)			
	N	Quartile 2	Quartile 3	Quartile 4
Primary Model ^1^	11,769	0.93 (0.77, 1.12)	0.60 (0.50, 0.74) *	0.55 (0.36, 0.84) *
Sensitivities				
+Controlling for Health Conditions ^1,2^	9039	0.86 (0.67, 1.10)	0.61 (0.48, 0.78) *	0.48 (0.31, 0.73) *
+Controlling for Lifestyle Behaviors ^1,3^	11,769	1.01 (0.84, 1.20)	0.69 (0.57, 0.84) *	0.70 (0.45, 1.07)
+Controlling for Day of Collection ^1,4^	11,769	0.93 (0.77, 1.12)	0.60 (0.50, 0.74) *	0.55 (0.37, 0.84) *
+Controlling for C-reactive protein ^1,5^	5377	1.02 (0.72, 1.44)	0.71 (0.54, 0.93) *	0.47 (0.25, 0.90) *

^1^ Estimated using logistic regression with robust standard errors. Models control for age, gender, race/ethnicity, educational attainment, relationship status, family poverty-to-income ratio, total caloric intake and time of year of dietary recall (November–April or May–October). + = additional model covariates. ^2^ Models additionally control for BMI, cardiac conditions, high cholesterol, hypertension, liver conditions, type 2 diabetes and cancer. ^3^ Models additionally control for current smoking status, recent alcohol use, and total minutes of moderate to vigorous physical activity over the past 30 days. ^4^ Models additionally control for control for day of the week of diet data collection. ^5^ Models additionally control for systematic inflammation, measured via C-reactive protein. * *p* < 0.05 comparing each quartile to quartile 1 (the lowest adherence to aMED).

**Table 4:** Heterogeneity in the association between Alternate Mediterranean Diet score (aMED) and moderate to severe depressive symptoms, NHANES 2007–2012.

	N	Mean Total aMED Score (SE)	Odds Ratio (95% Confidence Interval)
Age			
<30 years old	2032	3.13 (0.05)	1.02 (0.60, 1.75)
30–55 years old	5253	3.39 (0.05)	0.62 (0.49, 0.80) *
>55 years old	4484	3.83 (0.05)	0.46 (0.33, 0.64) *
BMI category			
Normal weight (18.5–24.99 kg/m^2^)	3127	3.72 (0.06)	0.57 (0.38, 0.86) *
Overweight (25.0–29.99 kg/m^2^)	3872	3.57 (0.06)	0.66 (0.43, 1.00) *
Obese (≥30.0 kg/m^2^)	4517	3.22 (0.03)	0.71 (0.55, 0.90) *

BMI = body mass index; SE = standard error. Estimated using logistic regression with robust standard errors. Models control for age, gender, race/ethnicity, educational attainment, relationship status, family income to poverty ratio, total caloric intake and time of year of dietary recall (November–April or May–October). * *p* < 0.05 comparing higher adherence to aMED to lower adherence to aMED, based on the median value for the total aMED score.

**Table 5:** Association between Alternate Mediterranean Diet score (aMED) and moderate to severe depressive symptoms, NHANES 2007–2012 using two 24-h diet recalls.

	Odds Ratio (95% Confidence Interval) ^1^		
	N = 9897		
	Quartile 2	Quartile 3	Quartile 4
Mean Value ^2^	0.61 (0.47, 0.78) *	0.65 (0.47, 0.90) *	0.49 (0.35, 0.69) *
Population Ratio Method ^3^	0.72 (0.56, 0.93) *	0.62 (0.45, 0.87) *	0.45 (0.32, 0.63) *
Bivarite Method ^4^	1.07 (0.85, 1.34)	0.95 (0.74, 1.23)	1.03 (0.84, 1.27)

^1^ Estimated using logistic regression with robust standard errors. Models control for age, gender, race/ethnicity, educational attainment, relationship status, family income to poverty ratio, total caloric intake and time of year of dietary recall (November–April or May–October). ^2^ aMED was calculated using the mean value for each component between day 1 and day 2 of dietary recall. ^3^ aMED was calculated using the population ratio method; this method uses the mean value between day 1 and day 2 of dietary recall and then estimates the ratios of the dietary constituents to 1000 kcal of energy, with the exception of fatty acids, which use the ratio of the sum of monounsaturated fatty acids to saturated fatty acids. ^4^ aMED was estimated using the bivariate method; this method uses adaptive Gaussian quadrature to predict usual intake for each individual. Predictive models include a random-effect and control for day of intake. * *p* < 0.05 comparing each quartile to quartile 1 (the lowest adherence to aMED).

## Data Availability

NHANES data is publicly available. The code and dataset used for the analysis are available upon request.
